# Artificial Intelligence and Data Science Methods for Automatic Detection of White Blood Cells in Images

**DOI:** 10.1007/s10278-025-01538-y

**Published:** 2025-05-16

**Authors:** Yawo M. Kobara, Ikpe Justice Akpan, Alima Damipe Nam, Firas H. AlMukthar, Mbuotidem Peter

**Affiliations:** 1 Odette School of Business, University of Windsor, Windsor, ON Canada; 2 Department of Information Systems and Business Analytics, Kent State University, 800 E. Summit Street, Kent, OH 44242 USA; 3 Residency Program, National Institute of Hygiene, University of Lomé, Lome, Togo; 4 Department of Information Technology, Catholic University in Erbil, Erbil, Kurdistan Region Iraq; 5 Healthcare Research Group, Ibom International Center for Research and Scholarship, Windsor, ON Canada

**Keywords:** Artificial intelligence, Generative artificial intelligence, Data science methods, Machine learning, Deep learning, White blood cells detection, Blood cells images segmentation, Leukemia, Blood disease, Diagnosing blood diseases processes

## Abstract

Data scieQuerynce (DS) methods and artificial intelligence (AI) are critical in today’s healthcare services operations. This study focuses on evaluating the effectiveness of AI and DS in biomedical diagnostics, including automatic detection and counting of white blood cells (WBCs) and types, which provide valuable information for diagnosing and treating blood diseases such as leukemia. Automating these tasks using AI and DS saves time and avoids or minimizes errors compared to manual processes, which can be complex and error prone. The study utilizes bibliographic data from SCOPUS to evaluate research on applying AI algorithms and DS methods for mapping and classifying WBC images for treatment of blood diseases, such as leukemia using literature survey and science mapping methodology. The results show the potency of different DS methods and AI algorithms, such as machine learning, deep learning, and classification algorithms that enable the automatic detection of WBC images. AI and DS algorithms offer critical benefits in effectively and efficiently analyzing microscopic images of blood cells. The automatic identification, localization, and classification of WBCs speed up the patient diagnosis process, allowing hematologists to focus on interpreting results. Automatic processes identify specific abnormalities and patterns, enhancing accuracy and timely diagnoses. Future work will examine the application of generative AI in blood cells diagnostics.

## Introduction

White blood cells (WBCs), or leukocytes, are the defensive force in the human immune system that protects the body against infections (e.g., viruses, bacteria, or fungal), inflammation, and other ailments [1, 2]. Most WBCs are manufactured in the red marrow of bones and special glands elsewhere in the body. Healthy humans possess about four to eleven thousand leukocytes in every cubic inch of blood [3, 4]. When the human body suffers an infection, signals are sent to the bone marrow and special glands to create more WBCs [1, 3, 4]. In addition to the defensive roles, WBCs promote tissue repair and contribute to overall human health [3, 4].

The past two decades have witnessed increased deployment of artificial intelligence (AI) systems and data science (DS) methods and algorithms for automatic detection of WBC [1, 2]. Accurate detection and counting of WBCs are essential to providing valuable information for diagnosing and treating blood diseases such as leukemia [1, 5, 6]. Adopting the AI and DS methods for WBC detection enhances performance efficiency and accuracy and minimizes or eradicates errors in WBCs’ counting [1, 7, 8]. WBC detection involves identifying and counting WBCs in blood samples [1, 5], which enhances the diagnosis of blood-related illnesses, including infections and blood disorders. Thus, determining the WBC count in human blood (often performed by a medical technologist) can indicate if someone has an infection [4, 5].

The WBC detection process combines several activities, including localization, segmentation, and classification of blood samples [1, 7, 8]. During medical diagnostics, the localization task involves identifying the approximate region or exact position of WBCs in blood samples, typically in a microscopic blood smear image [9]. Other critical steps in the WBC detection process include segmentation and classification. The segmentation task explains the separation of WBCs from other blood components, such as red blood cells, platelets, and other particles in a blood smear image [5, 6]. The classification task involves stratifying the WBCs into different types [1, 6].

The past two decades have witnessed increased deployment of AI and DS methods in biomedical diagnostics, particularly in performing WBC detection activities and tasks, including localization, segmentation, classification, and WBC counting, offering performance efficiency and accuracy [7, 8, 10–13]. Automating these tasks with AI and DS saves time and avoids or minimizes errors compared to the cumbersome and error-prone manual processes [10]. This study evaluates the use cases and effectiveness of the AI and DS methods and techniques for automatic detection of WBC images, which helps to treat blood related ailments such as leukemia patients. The research seeks to achieve the following three specific objectives:

- RO1. Analyze the research trends of AI and DS applications in WBCs research and sources citation impact analyses.
- RO2. Evaluate the research landscape, hotspots, and evolutionary trends of research on AI and DS applications in automatic detection, classification, and segmentation of WBC.
- RO3. Identify the use cases and map specific AI and DS techniques/algorithms for WBC detection and the types of health conditions addressed.
- RO4. Assess the effectiveness of AI and DS methods for WBC detection.

The rest of the paper is organized as follows: the “Background” section presents an overview of WBCs’ biomedical properties and functions and the theoretical backgrounds of AI and DS algorithms. The “Materials and Method” section describes the materials and methods. The “Results and Discussion” section presents and discusses the results. Finally, the “Conclusion and Recommendations for Future Research” section concludes the paper and recommends areas for future research.

## Background

### Overview of WBCs

The WBCs are blood cells that aid in the body’s defense against infection [3, 4]. Clinicians specializing in evaluating blood and blood disorders are known as Hematologists. These specialists can diagnose, treat, and manage conditions that affect the blood and the components of the blood, including red blood cells, WBC, platelets, blood vessels, bone marrow, lymph nodes, and spleen. Hematologists can identify, classify, and count WBC and understand the distinct characteristics, such as size, morphology, and specific proteins or granules. They are a diverse group of nucleated cells that circulate in the bloodstream. The average concentration in the blood of an adult typically ranges between 4000 and 11,000 per microliter [3, 4]. When the WBC concentration is below 4000 WBC/ml, a person is said to have leukopenia, while if it is above 11,000, it is termed leukocytosis [3–6].

### Biomedical Properties and Functions of WBCs

Generally, WBCs are classified into two groups: granulocytes and agranulocytes. Granulocytes consist of three types (neutrophils, eosinophils, and basophils) that form part of the innate immune system and are responsible for immediate defense against pathogens that may attempt to invade the human body [14–16]. Also, the granulocytes are characterized by granules within their cytoplasm, which contain various substances essential for the immune system’s response to infection, inflammation, and other immune challenges [14, 15]. On the other hand, agranulocytes do not contain granules in their cytoplasm. However, the agranulocytes also play crucial functions in the body’s immune system by fighting infections and removing debris (dead cells and tissue debris) [15, 16]. It has two main sub-categories (lymphocytes and monocytes) [16, 17]. Table 1 presents the WBC types and functions.

**Table 1:** WBC classification, types, and functions

Group	White blood cell types and functions
Granulocytes	**Neutrophil**: A type of WBC that forms the essential part of the immune system that fights microorganisms, particularly bacterial infections, by ingesting and destroying the invaders with enzymes [18, 19]
	**Eosinophils**: Another type of WBC with the main function of controlling inflammation, especially active during parasite infections and allergic reactions, stops substances or other foreign materials from harming the body [18]. Compared to neutrophils that may have 2 to 5 lobed nuclei, eosinophils only have a bi-lobed (two lobes) nucleus that is shaped like a horseshoe. They will also appear spherical with fine granules referred to as acidophilus refractive granules [17]
	**Basophils**: This is a type of granulocyte and immune system that fights inflammation and allergic reactions, releasing substances like histamine and heparin. It is the least common type of WBC and constitutes a fraction (less than 1%) of circulating leukocytes [18]. Compared to the other granulocytes, basophils have a large and irregular nucleus that resides inside the spherical shaped cell. Whereas the nucleus of the other granulocytes is well defined and can be clearly described, the nucleus of a basophil (bluish under the microscope) is large and irregular inside the cell and may prove difficult for students to describe. Basophils are responsible for producing enzymes during asthma attacks and allergic reactions
Agranulocytes	**Lymphocytes**: These are agranulocyte-type WBCs responsible for the immune system and consist of the B cells (for antibody production) and T cells found in blood and lymph tissue (for regulating immune response). While they are smaller compared to other leukocytes, lymphocytes have a large round nucleus that takes up much of the cell volume. As a result, lymphocytes have very little to no cytoplasm and use antibodies to stop bacteria or viruses from entering the body (B-cell lymphocyte) also kills off the body’s cells if they have been compromised by a virus or cancer cells (T-cell lymphocyte) [20]
	**Monocytes**: This is another subtype of WBCs that form part of the immune system, primarily patrolling the body for pathogens, fighting infections, and initiating immune responses [20]. Compared to lymphocytes, monocytes are larger with a nucleus that is a bean or kidney shaped. These cells also have more cytoplasm compared to lymphocytes. It becomes a macrophage in the body’s tissues, ingesting microorganisms and getting rid of dead cells while increasing immune system strength

Neutrophils are the most common type of WBCs (50–80% of WBCs) and play a critical role in the innate immune response. They are highly effective in phagocytosis, engulfing and destroying bacteria, fungi, and other pathogens [21]. They are produced in the bone marrow and have a multilobed nucleus, with two to five lobes separated by thin filaments. They circulate in the blood and migrate to the tissues to carry out their functions. Their lifespan in circulation is relatively short, around 7–10 h, while in tissues, it ranges from 1 to 2 days [22]. Neutrophils carry out a variety of tasks, including migration along the vessel wall (margination), adhesion to the endothelium (lining of blood vessels), transcellular and paracellular migration through the endothelium (diapedesis), movement through tissues in response to chemical signals (chemotaxis), engulfment and internalization of pathogens (phagocytosis), killing and digestion of microorganisms, as well as present evident morphological characteristics and functions of eosinophils [22–25]. Generally, eosinophils (1–4% of WBCs) combat parasitic infections and allergic responses. They release toxic granules to kill parasites and participate in the regulation of inflammation and allergic reactions. They are slightly larger than neutrophils. The nucleus is usually bilobed. Eosinophils have acidophilic granules, which are larger than those of neutrophils. In addition to granules, the weakly basophilic cytoplasm contains abundant glycogen particles and more numerous and more extensive mitochondria that are found in neutrophils; there is also some rough endoplasmic reticulum. The eosinophil lifespan is about 1 day in circulation and 8–12 days in the tissues [23–25].

Eosinophils synthesize and secrete growth factors, cytokines, and chemokines and regulate innate and adaptive immune responses and tissue remodeling and repair. These WBCs also trigger histamine release by basophils and mast cells and are essential in controlling infection by multicellular parasites. They are attracted to the sites of parasitic infection by chemokines such as eutaxia 1 and 2 and, at such sites, are activated by cytokines produced by helper T cells [20]. They attack parasites by degranulation and peroxidase-mediated generation of reactive oxygen species. Eosinophil neurotoxin and ribonuclease have antiviral properties. In addition to these valuable functions, eosinophils help in maladaptive allergic conditions.

Huntley [24] also comprehensively reviews basophils’ functions and morphological characteristics. Basophils (0–1% of WBCs) are involved in allergic responses and immune regulation and release histamine and other mediators during allergic reactions, contributing to inflammation and immune response modulation. The granulocytes possess large dark purple granules that almost obscure the nucleus. The nucleus is usually bilobed. Basophils can survive many days in circulation. Cytoplasm plays key roles in protection against helminth infections but is also involved in allergies, anaphylaxis, and chronic inflammation; they secrete histamine, serotonin, heparin, and proteolytic enzymes [24].

Agranulocytes are WBCs characterized by the absence of visible granules in their cytoplasm [18–20]. Unlike granulocytes, which have granules that contain various substances important for immune responses, agranulocytes have a smooth and uniform appearance under the microscope. There are two main types of agranulocytes: the lymphocytes and the monocytes. Lymphocytes (20–40% of WBCs) provide adaptive immunological responses. They stand out by their diminutive size, spherical form, and sizable nucleus encircled by a thin ring of cytoplasm and subdivided into T, B, and natural killer (NK) cells [20]. B cells create antibodies for humoral immunity, T cells are important for cell-mediated immunity, and NK cells are essential in targeting infected or aberrant cells [24]. Monocytes (2–10%) are the largest peripheral blood cells. They circulate in the bloodstream and migrate to tissues, where they differentiate into macrophages and other specialized reticuloendothelial system cells and into dendritic cells and osteoclasts. They are essential for phagocytosis, antigen presentation, triggering immune responses, and killing microorganisms (including mycobacteria, Listeria, and fungi). These are immune modulators, secreting IL-1, IL-6, IL-12, tumor necrosis factor-a, interferon-a, and interferon-b when stimulated, thus enhancing the inflammatory response [18–20, 26, 27]. Their intravascular lifespan is 1–3 days, while the cells into which they differentiate are long-lived. Macrophage functions include the removal of unicellular parasites from erythrocytes; removal of Howell-Jolly bodies and other red cell inclusions; removal from the circulation of senescent red cells; phagocytosis of other senescent or dead cells; storage of iron as ferritin and hemosiderin; and supply of iron to developing erythroblasts. A complete exposition is available elsewhere [20, 28].

**Fig. 1:**
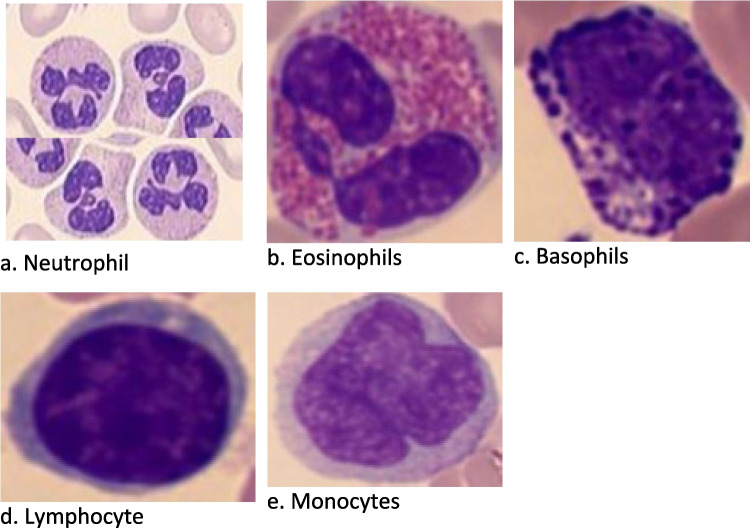
White blood cell types (**a**–**e**). Images adapted from unpublished doctoral thesis by Al-Mukhtar [17], a co-author in this article

Each type of white blood cell plays a specific role in the immune system’s defense mechanisms, contributing to the body’s overall protection against infections, foreign substances, and abnormal cells. It is important to note that this classification represents the main types of WBCs. Some sources may categorize them differently or include additional subtypes based on specific criteria or context. Figure 1a–e shows the five types of WBCs.

WBCs are involved in various immune functions, including phagocytosis (engulfing and destroying pathogens) and immune response, contributing to the defense against infections. Identifying, classifying, and counting WBCs are essential for assessing overall health and diagnosing various medical conditions. The white blood cell count provides valuable information about the immune system and helps healthcare professionals in several ways. First, it helps detect infections; an increase in neutrophils number can indicate the presence of infection since they are the first responders to bacterial infections, and their increased numbers suggest an active immune response. Secondly, it helps monitor inflammatory conditions such as rheumatoid arthritis, vasculitis, or inflammatory bowel disease; thirdly, low white blood cell counts (leukopenia) are often associated with bone marrow disorders, chemotherapy, or certain viral infections.

Conversely, high white blood cell counts (leukocytosis) may indicate leukemia, myeloproliferative disorders, or other blood-related conditions. Thus, patients undergoing cancer treatment, such as chemotherapy, may experience changes in their white blood cell counts, and regular monitoring helps healthcare professionals adjust treatment plans and manage side effects, such as increased susceptibility to infections. Finally, certain autoimmune disorders, like lupus or vasculitis, can also affect the white blood cell count. Monitoring changes in the count over time can aid in managing these conditions. Hematologists can use automatic white blood cell recognition from microscopic pictures to help them diagnose a range of blood illnesses. Automatic white blood cell recognition can support diagnostic decisions by analyzing the morphological properties of WBCs [29].

### AI and DS Method Application in Biomedical Diagnostics

The recent advances in computing processing power and the introduction of advanced technologies such as AI, big data analytics, and other data science (DS) methods offer significantly potent tools for solving diverse healthcare problems, biomedical engineering operations, and clinical diagnostics [7, 10, 26]. Some recent studies identify medical and healthcare operations as leading users of AI technology and DS methods [30–32].

AI is a technological innovation that focuses on creating applications and systems to perform tasks that usually require human intelligence [33]. Thus, the purpose of AI is to mimic human thinking and behavior in problem-solving, decision-making, and language understanding [33, 34]. The AI technology encompasses many techniques, including rule-based systems, knowledge-based systems, expert systems, and statistical learning methods. It is not limited to data-driven approaches but includes logic-based reasoning and symbolic processing [33]. The latest development in the AI domain witnesses an integration of advanced technological capabilities, including conversational agents like ChatGPT and other generative AI (GenAI) applications, which further enhance human-like thinking and problem-solving in dynamic environments [35].

On the other hand, DS is a multidisciplinary field that utilizes diverse methods and techniques in mathematics, statistics, and computer science to solve problems involving gleaning insight from structured and unstructured data [11, 12, 30]. Some of the DS methods include machine learning (ML), deep learning (DL), data mining, data visualization, and predictive modeling to solve real-world problems using data-driven approaches [30]. These sophisticated data analytics techniques enable analysts to uncover patterns in complex data and offer solutions to complex real-world problems [12, 30, 35].

The above explanations of AI and DS concepts highlight subtle differences and similarities between both technologies. First, some aspects of AI and DS use similar analytics methods, such as ML, DL, data mining, and more. ML can be defined as an algorithm developed to allow computers to learn from data and improve task performance without being explicitly programmed [17, 21, 36]. However, unlike the traditional AI approaches that rely on predefined rules, ML enables systems to discover patterns and make data-driven predictions. Thus, ML is a data-driven approach under AI. Some of the traditional ML methods include regression, decision trees, support vector machines (SVMs), and random forests [17, 21, 37].

DL is a subset of ML that uses artificial neural networks with multiple layers (deep neural networks) to model complex patterns in large datasets. It is particularly effective for tasks involving high-dimensional and complex unstructured data, such as images, speech, and natural language [9, 36]. DL is well suited for identifying patterns in medical image analysis, such as WBC images [9]. DL has several approaches depending on the structure of the neural networks and the kind of data to handle, including convolutional neural networks (CNNs), recurrent neural networks (RNNs), and more. CNNs, which relate to this study, are DL models designed for image processing tasks. Its typical architecture makes it possible to extract spatial features from WBC images using filters (kernels) and performing final classification based on extracted features [38]. It is highly effective for feature extraction and thus can automatically learn morphological differences in WBCs. However, it requires large, labeled datasets and high computational power. RNNs, as a DL approach, can be utilized to process sequential data, such as text, speech, or time series [38].

SVMs are supervised learning models that classify data by finding the optimal hyperplane that separates different classes. It transforms input features into a higher-dimensional space where they are more separable and optimizes the margin between different WBC classes. Unlike CNNs, SVMs require fewer samples for effective training and work best with handcrafted features [39].

Random forest is another ML technique, also known as an ensemble learning method, that consists of multiple decision trees [40]. The key components include an aggregation of bootstrapping called bagging that generates multiple decision trees trained on different subsets of the dataset. Each tree considers a random subset of features to reduce correlation. The final classification is based on the majority vote from all trees.

The AI and DS technologies make up a disruptive force for managing healthcare operations and processes, which can significantly improve disease diagnoses, patient care, and efficient patient records management [7, 10, 41, 42].

## Materials and Method

### Data Collection

The data used for this research came from publications collected from the SCOPUS bibliographic database. A reliable and comprehensive database is essential for conducting a systematic literature review. The rationale for selecting the SCOPUS bibliographic database for this study was its extensive coverage, high-quality indexing, and relevance to AI and DS-based biomedical research. Existing literature recognizes SCOPUS as one of the largest abstract and citation databases covering multidisciplinary disciplines with over twenty-five thousand peer-reviewed journals, including computer science, artificial intelligence, biomedical engineering, and medical imaging journals [43]. This interdisciplinary coverage is essential for this study. The database is also known for its robust indexing system, which ensures that only quality sources are included [43]. The SCOPUS bibliographic data source also tracks citations across disciplines, helping to identify the most influential research papers in AI-based WBC classification. The h-index and citation metrics available in SCOPUS help evaluate the impact of studies, enabling the selection of the most relevant and significant research. Also, the Boolean search capabilities, allow precise keyword searches (e.g., “deep learning,” “white blood cells,” “detection,” or “classification”). Further, users can filter bibliographic data by document type, publication year, and subject area, ensuring that only relevant documents relating to biomedical studies are collected. The availability of file export features in different formats (text, csv—comma separated value, Microsoft Excel, etc.) facilitates the filtering/screening processes and analysis.

The first literature search and data collection occurred in early December 2023. However, the final survey update took place on December 31, 2023. However, revisions were made to the query string during revisions following reviewers feedback leading to an increase in the sample size. Table 2 presents the search terms, keywords, and phrases used to create the query string for the data retrieval.

**Table 2:** Articles’ search, filtering, screening, and selection criteria

Activities/focus	Criteria
Search term and query string	TOPIC: ((“data science” OR “Deep Learning” OR “Artificial Intelligence” OR “Natural Language Processing” OR “Data Warehousing” OR “Data Mining” OR “Data Visualization” OR “Machine Learning” OR “Algorithm”) AND (“White blood*” OR “leukocyte” OR “leukemia”) AND (“auto* classification” OR “auto* detection” OR “wbc” OR “auto* segmentation”)). The search generated 329 published documents
Database source	SCOPUS
Publication years	Period covered/searched: all years; effective years: 1995–2023

### Filtering, Screening, and Selection of Publications

**Fig. 2:**
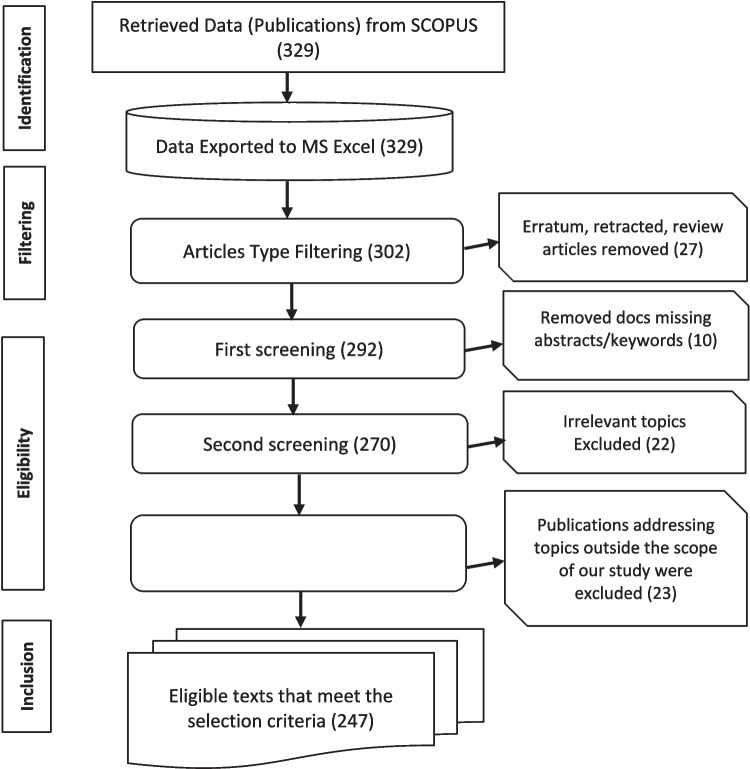
PRISMA process for filtering, screening, and selection of publications/data collection

The filtering and selection of the publications follow the preferred reporting items for systematic reviews and meta-analyses, commonly known as PRISMA [44]. Adopting the PRISMA processes in the filtering, screening, and selecting publications used in the study enhances clarity, transparency, and completeness in selecting the publications from which we retrieved the data used for the analysis in this study [44]. The PRISMA technique also enhances the reproducibility of the results. The filtering, screening, and selection processes involve scanning and querying the title and abstract of the publications. Figure 2 presents the PRISMA processes used in this study.

To ensure the quality of publications included in this study, we subjected the identified published documents through further filtering, screening, and selection processes involving scanning and querying the title and abstract of the published documents. As shown in Fig. 2, the process of data filtering, screening, and selection of the sample follows the PRISMA methodology. After the bibliographic database search, the data was extracted as a.CSV (comma separated value) file and exported to Microsoft Excel 365 version for screening. The filtering process involved removing the non-peer-review publications, including retracted/erratum papers, and reviews, totaling 27 documents. Next, we removed ten (10) publications that did not contain abstract and twenty-two (22) irrelevant documents that did not address the topics of interest. The final stage of the screening involved scanning the abstracts to ensure that the articles addressed the topic on the use of AI and DS methods to address WBC detection. For example, publications on WBC detection, segmentation, and counting using non-AI and non-DS methods, such as electromagnetism optimization algorithm, evolutionary algorithm, or colony bees’ technique, were removed. Similarly, studies that utilized the AI and DS methods for other classification purposes, such as red blood cells (RBC), were outside the scope of this study and were excluded.

### Science Mapping and Analysis

A total of 247 documents were screened (Fig. 2) and exported as comma separated value (.csv) onto R-Studio Programming environment for science mapping, quantitative analysis, and descriptive narratives. The analysis involved science mapping of AI and DS techniques, and different WBC tasks as presented by selected publications. The analyses establish the utilization of AI and modern DS techniques for automatic detection, classification, and segmentation of WBCs. The study utilizes an open-source bibliometric analysis package, Bibliometrix, embedded in the R-Studio [45, 46], and VOSviewer [47].

## Results and Discussion

### Scientific Literature Productivity Trend

**Fig. 3:**
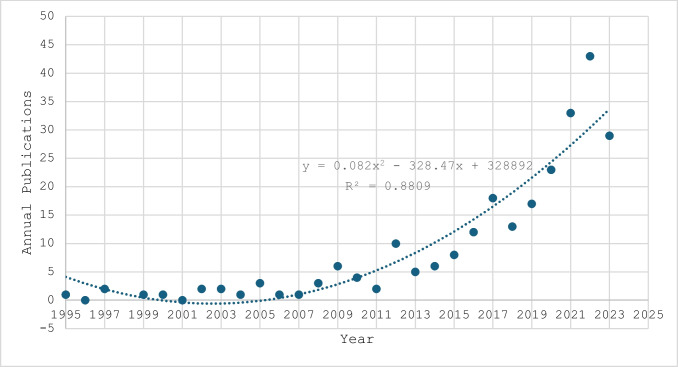
The scientific literature publications on artificial intelligence and data science methods for automatic detection of white blood cells for leukemia patients

**Fig. 4:**
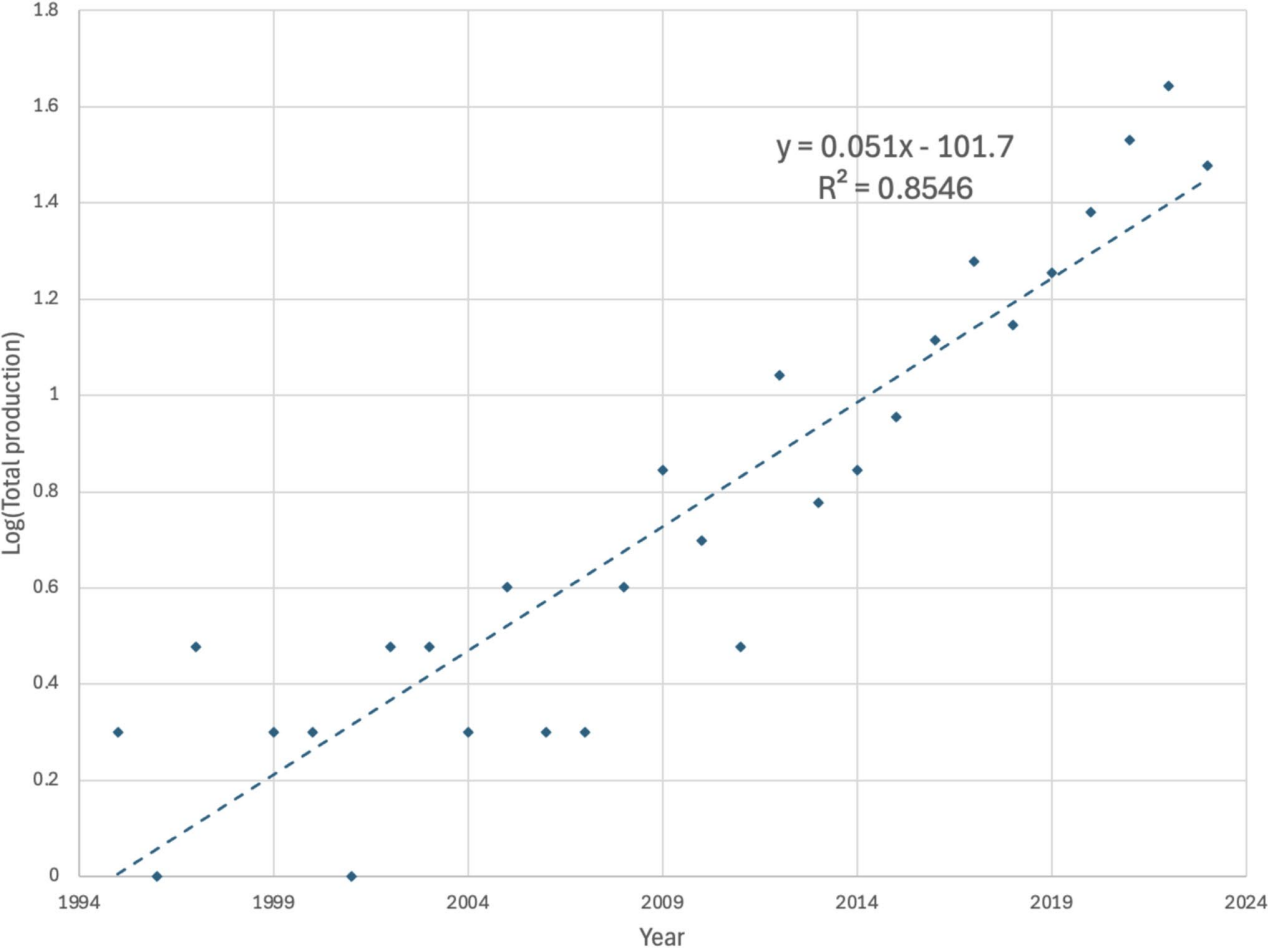
Log scale of the scientific literature publications on artificial intelligence and data science methods for automatic detection white blood cells for leukemia patients

The data for the analysis came from the 247 selected publications extracted from SCOPUS in text (.txt) and (.csv) formats and exported to Excel and the Bibliometrix application embedded in the R-studio programming environment for science mapping and quantitative analysis. The publications occurred between 1995 and 2023. Figures 3 and 4 show the publication trend during the period. The literature productivity trend and the citation impact analysis address the first research objective (RO1).

The result (Fig. 3) shows that there were just a few publications on the use of AI and DS methods for WBC detection, segmentation, and classification in the 1990 s. The few publications on the topic of interest are not surprising as these early periods did not experience modern DS techniques. However, the period 2000–2009 shows a slow but steady increase in the number of articles produced, totaling 24 by 2010. The pre-COVID period (2011–2019) shows an increase in annual publications on the applications of AI and DS in WBC detection as these technologies garnered interest in the biomedical diagnostics domain.

The result (Figs. 3 and 4) shows that there were just a few publications on AI and DS methods for WBC detection, segmentation, and classification in the 1990 s. The few publications on the topic of interest are not surprising as these early periods did not experience modern DS techniques. However, the period (2000–2010) shows a slow but steady increase in the number of articles produced, totaling 24 in this period segment. The pre-COVID years (2011–2019) showed an increase in annual publications on the applications of AI and DS in WBC detection as these technologies garnered interests in the biomedical diagnostics domain.

The period from 2020 corresponds with the COVID-19 outbreak, which recorded significant growth in the application of AI and DS methods in healthcare generally, and specifically for automatic detection, segmentation, and classification of COVID-19 and non-COVID-19 related pneumonia symptoms, comorbidities, and other blood-related diseases [48–50]. Thus, the annual publications in this domain surged remarkably from an average number of 9 documents per year and peaked at 43 published articles in 2022.

This increase is not surprising, considering that severe coronavirus disease pandemic cases led to blood clots in several patients [49–51]. The surge in the number of articles during and after COVID-19 is a pointer to the role of data science in preparedness for future pandemics. Many multidisciplinary collaborations among scientists, researchers, and institutions globally open the sharing of ideas, data, and resources, bringing together experts from virology, immunology, epidemiology, data science, and other fields. This interdisciplinary collaboration also impacted research output between 2020 and 2023 [51, 52].

Figure 4 presents a scatter plot of the scientific publications (SP) for the period to visualize the raw trend, which fits an exponential curve. Since there are some years with zero count, instead of modeling log(*y*), we modeled log(*y* + 1). The exponential model produced the regression equation (log(*y* + 1) = 0.051*x* − 101.7), where *x* is the *n*th year and *y* is the logarithm of the total number of SP.

As shown in Figs. 3 and 4, we developed two regression models to analyze the variation in the annual SP. The first predictive model assumes a polynomial function of degree two (Fig. 3). The second is the exponential model based on the logarithm transformation of the SP. Using Microsoft Excel 365 and the yearly publication data, we created the regression model using the data analytic option. The quadratic model produced the regression equation (*y* = 0.08*x*^2^ − 328.47*x* + 328,892), where *x* = *n*th year. The exponential model produced the regression equation (log(*y* + 1) = 0.051*x* − 101.7), where *x* is the *n*th year and *y* is the logarithm of the total SP.

The results (Figs. 3 and 4) show that time is a good predictor of variation in SP (annual increases). The quadratic model has an *R*^2^ = 0.88, and the exponential model has an *R*^2^ = 0.85. The quadratic model implies that 88% of the variation in the yearly SP is explained in the regression model (*y* = 0.08*X*^2^ − 328.47*X* + 328,892). On the other hand, using the logarithmic model shows that 85% of the variation in the yearly SP is explained in the regression model (log(*y* + 1) = 0.051*x* − 101.7). Although the coefficient of the determination of the quadratic model is higher, the logarithmic model is more appropriate for this dataset because the trend of the SP over time exhibits accelerating growth, particularly after 2010 (Figs. 3 and 4). The publications pattern, especially in rapidly emerging or popular fields, often experience such exponential increases due to growing research interest, funding, technological advances, and network effects among researchers [32]. A quadratic model could inadequately represent this pattern by eventually predicting unrealistic deceleration or decline, while an exponential model captures the ongoing rapid acceleration more accurately. Therefore, the logarithmic model is selected as the model that best describes the SP trend.

### Eminent Sources and Impact

Table 3 shows the top ten relevant and eminent sources and the corresponding number of publications addressing the topic of automatic detection, classification, and segmentation of WBCs using AI and DS methods. The results of the bibliometric analysis using “R-Studio Bibliometrix” [45] identify 164 sources that published the 247 documents reviewed in this study. However, the top ten listed sources published 46 or 19% of the scientific publications. The most eminent source (Lecture Notes in Computer Science [Includes Subseries Lecture Notes in AI and Bioinformatics]) published the most articles (8), followed by “SPIE Proceedings—International Society for Optical Engineering,” which published six documents. The document types show that 63% of published documents are journal articles, 27% are conference papers, and the remaining 10% are book chapters. As expected, most sources are in the computer science and biomedical genre, and conference proceedings are in related fields.

**Table 3:** Journals in which articles were published

No	Element	NP	Citations	PY_Start	H_Index*
1	Lecture Notes in Computer Science (Includes Subseries Lecture Notes in AI and Bioinformatics)	8	56	2008	470
2	SPIE Proceedings—The International Society for Optical Engineering	6	5	2000	193
3	Medical and Biological Engineering and Computing	5	275	2012	111
4	Biomedical Signal Processing and Control	5	202	2021	108
5	Computers In Biology and Medicine	5	89	2021	125
6	Multimedia Tools and Applications	4	83	2022	116
7	Cancers	4	59	2021	133
8	Computational Intelligence and Neuroscience	3	235	2022	88
9	Computational and Mathematical Methods in Medicine	3	153	2013	78
10	Advances In Intelligent Systems and Computing	3	119	2013	69

*NP*, number of publications; *PY_Start*, publication start year; H-Index*, sources: www.scimagojr.com

The top ten sources are high-impact journals and proceedings (based on the H-index measure). For example, “Lecture Notes in Computer Science (includes Subseries Lecture Notes in AI and Bioinformatics)” has the highest H-index of 470 among the eminent sources. Table 3 presents the complete list.

### Documents’ Citation and Impact

Table 4 presents the top ten most cited scientific publications and research themes that apply AI and DS methods for automatic detection, segmentation, and classification of WBCs. The most cited paper examines the automatic segmentation of clustered nuclei using image segmentation algorithms [53]. In this paper, the author argued that the strategies in the two main algorithms presented are based on cluster division by detecting internuclear gradients or division of domains of influence. The paper introduces novel DS algorithms or approaches for the general analysis of cells, and the methods prove to be more accurate, efficient, and versatile than existing techniques.

**Table 4:** Most cited papers and research themes

	**References**	**Research themes**	**Total citations**	**TC per year**	**Normalized TC**
1	Malpica et al., 1997 [53]	Application of ML algorithms	399	14.78	1.94
2	Hegde et al., 2019 [54]	Classification of white blood cells in peripheral blood smear images	121	24.20	4.21
3	Cintuglu et al., 2016 [55]	Architecture of autonomous systems	72	6.00	3.16
4	Mohamed et al., 2012 [56]	An efficient technique for white blood cells nuclei automatic segmentation	71	10.14	3.53
5	Klomsae et al., 2017 [57]	Unsupervised detection algorithm	71	3.74	1.18
6	Saraswat et al., 2013 [58]	Leukocyte segmentation in tissue images using differential evolution algorithm	66	6.00	2.28
7	Loey et al., 2022 [59]	Deep transfer learning in diagnosing leukemia in blood cells	59	14.75	4.72
8	Begg, 2008 [60]	WBC counting and probability measures	49	2.58	0.82
9	Saraswat et al., 2014 [40]	Feature selection and classification of leukocytes using random forest	48	4.80	2.11
10	Gautam et al., 2016 [61]	Automatic classification of leukocytes using morphological features and naïve Bayes classifier	47	6.71	2.34

The next most cited paper [54] created an optimal automated classification model of WBCs using the decision support system. The paper demonstrates the automatic classification of WBCs’ images and compares traditional image-processing approaches and deep learning methods for WBCs’ classification. In addition, the authors evaluated neural network classifier results for hand-crafted features and obtained an average of 99.8% accuracy. The article offers a direct implication for clinical practice. Problems faced generally involve large-scale studies or comprehensive datasets needed to provide valuable insights and model validation. Other most cited articles and themes are listed in Table 4.

### Research Hotspots, Visualization, and Trends of AI and DS Application in WBC Auto Detection

This section employs big data analytics software Bibliometrix embedded in R-Studio [45] and VOSviewer [46, 47], as explained in the “Materials and Method” section, to map the research themes and visualize the evolutionary trends of its thematic structure in a network map. The text analytics also highlights the research topics and trends and identifies the research domain, the most researched topics, and the period of occurrence. The results help to address the second research objective (R02), evaluating the research landscape, hotspots, and analyzing the evolutionary trends of research on AI and DS applications in automatic detection, classification, and segmentation of WBC.

**Fig. 5:**
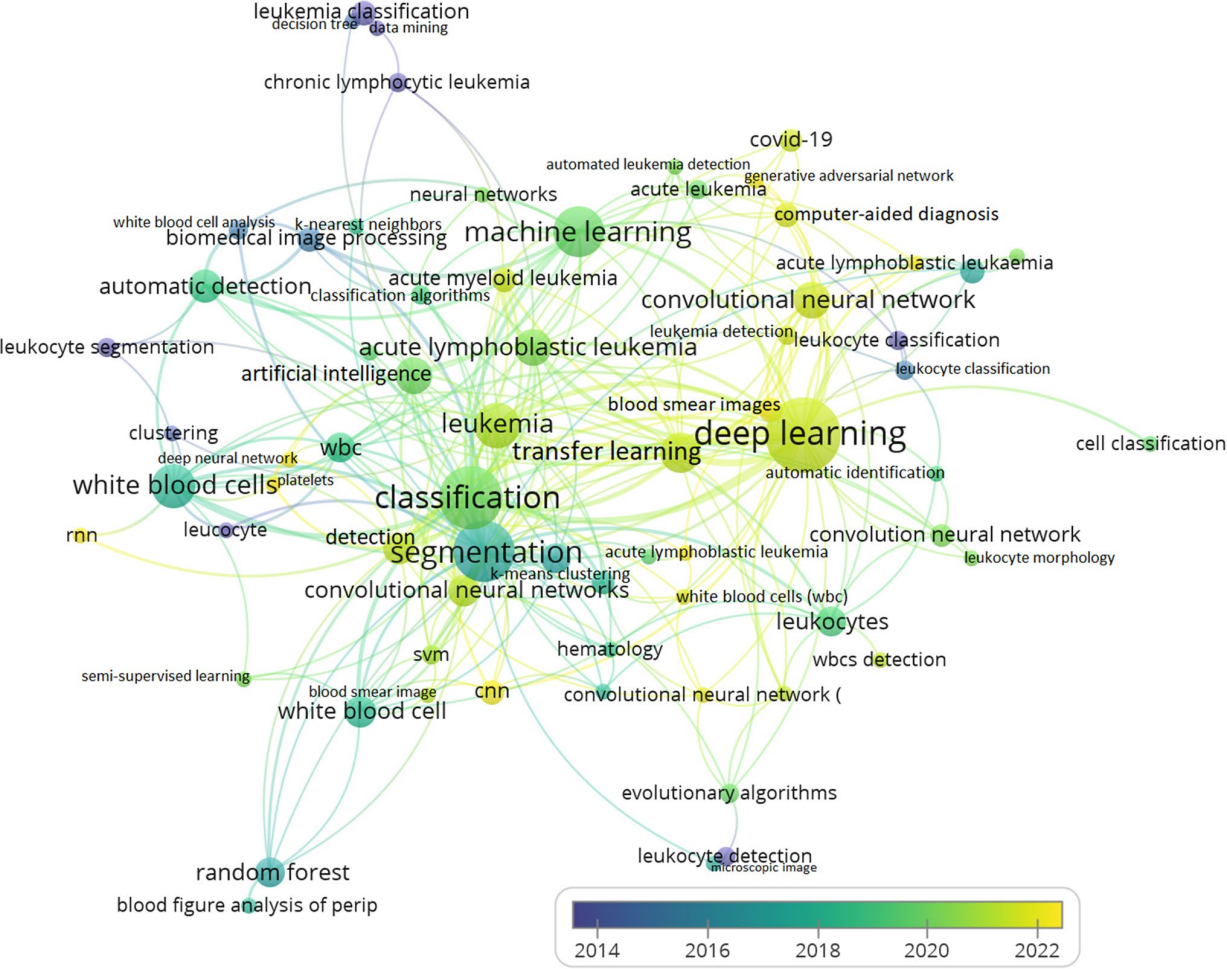
Network visualization of research themes trends on AI and DS methods for automatic detection of WBCs. Themes that occurred pre-2014 are in purple, between 2014 and 2016 are in blue, between 2016 and 2018 are in green, between 2018 and 2022 are in lemon-green, and post 2022 are in yellow

The dataset contains a total of 592 unstemmed unique author keywords. The keywords serve as a pointer to the research landscape [62]. The results of the text analytics using the VOSviewer application (Fig. 5) show that about 20% of the unstemmed unique keywords (118 out of 592) occurred twice or more in the dataset, implying word frequency of 2 and above (*f* ≥ 2), meaning that at least the keywords appeared in two or more studies. The text analytics algorithm categorizes the keywords into color-coded clusters. Also, the themes in the same Cluster are mapped to the colored trend to correspond with the cluster colors marked by the year of publication. The keywords are not stemmed, meaning that terms with similar meanings or words spelled differently are considered unique (e.g., “leukemia” and “leukemia” are considered unique terms). Figure 5 presents a complete network visualization map. The circles (nodes) represent the keywords/terms, while the size of the circles implies the word frequency (number of times that each word/term appears in the dataset). On the other hand, the links/lines, also called edges that connect the nodes (circles), represent the relatedness among the keywords. The keywords are color-coded, implying that circles with the same color belong to the same Cluster as shown in Fig. 5.

The top three most frequent keywords include “deep learning: 44,” “classification: 31,” and “white blood cell(s)/WBC: 30.” The next most frequent terms were (“machine learning”: 29; “segmentation”: 29; “leukemia: 16” (as a single word). Others used the terms such as “acute leukemia.” Other relevant keywords in this study were “artificial intelligence” and WBC “detection,” appearing 11 and 8 times, respectively. The themes are classified into five color-coded clusters highlighting the evolutionary trends and research focus that employ AI and DS techniques for WBC detection, classification, and segmentation.

The node labels represent the research themes under focus in each period segment mapped to the period of occurrence or year of publication (pre-2014, [1995 and 2014]; 2014–2016; 2016–2018; 2018–2020; 2020–2022; and post-2022 being the current era).

1. *Purple*: The themes in this Cluster cover the period before 2014. Some of the terms in the purple-colored Cluster include “leukemia,” “leukocyte classification,” “leukemia classification,” “leukocyte detection,” and more.
2. *Blue*: The themes in this Cluster were published between 2014 and 2016 (some of the items are “biomedical image processing,” “white blood cell analysis,” “decision tree,” and more.
3. *Green:* Published between 2016 and 2018 (“machine learning,” “artificial intelligence,” “automatic detection,” “classification,” and more).
4. *Lemon-green*: Occurred between 2018 and 2022 (“leukemia,” “transfer learning,” “deep learning,” and more).
5. *Yellow*: This Cluster contains the most recent themes published between post-2022 (“cnn: convolutional neural networks,” “computer-aided diagnosis,” “rnn: recurrent neural network,” “deep neural network,” “generative adversarial network,” “blood smear images,” and more.)

The themes dynamics (1–5 above) identify the trends and evolution in applying AI and DS methods in WBC detection studies. Some research themes appeared in more than one segment period (a–f). For example, a WBC detection study was repeated in different periods using a different technique. The research trends closely mirror developments in computer science advances and information development in industry practice. While the early years of research focused on diagnosing leukemia using WBC image smears [6, 63, 64], more recent years highlight technological trends using DS methods, such as machine learning methodology and “generative adversarial networks” [65]. The above results provide a historical overview and evolutionary trends in WBC detection research studies, highlighting the integration of AI and DS methods in biomedical services operations over the years.

### Use Cases of AI and DS Methods for WBC Detection, Segmentation, and Classification

This section identifies and maps sample use cases of AI and DS techniques and algorithms for automatic detection, segmentation, and classification of WBCs, which helps in diagnosing health conditions. The results address the third research objective (RO3).

Table 5 presents the results of the science mapping study highlight the various DS and AI models and algorithms employed to analyze patient blood to predict the presence of a particular medical condition. For example, the identification of leukemia and the detection of abnormal cells in peripheral blood was done [66]. The effectiveness is assessed based on accuracy, efficiency, scalability, clinical application potential, and robustness. The discussion evaluates the various AI/DS techniques commonly applied for WBC classification. Table 5 summarizes the WBC classification problems and applicable health conditions mapped to the AI/data science, the related technique/algorithm, and the problem description.

**Table 5:** AI and DS methods and WBC detection and classification problems tackled

Applicable health condition	AI/data science technique/algorithm	Description	Authors
Acute lymphoblastic leukemia (ALL)	Convolutional Neural Networks (CNN)	CNNs are used for automatic classification of leukemic cells from blood smear images, allowing for rapid diagnosis of ALL	[38, 54, 67]
Differentiating leukemic and non-leukemic cells	Random forest	This technique is utilized for feature-based classification of WBCs to distinguish between leukemic and non-leukemic cells	[37, 40]
ALL classification	Transfer learning	Adapting pre-trained models for improved classification of ALL based on limited data	[59, 67, 68]
Acute myeloid leukemia (AML)	Deep learning (DNNs, CNNs)	Deep learning models automate the classification of AML cells, improving accuracy in identifying AML subtypes	[38, 69–71]
Detecting WBC textural features	K-nearest neighbors (KNN)	KNN is used to classify WBCs based on morphological and textural features to detect AML	[53, 72, 73]
Chronic lymphocytic leukemia (CLL)	Support vector machines (SVM)	SVM is employed to classify WBC types and identify CLL from flow cytometric immunophenotyping data	[74, 75]
Classifying CLL	Decision trees	Decision tree algorithms help in interpreting complex data sets to classify CLL based on various features	[76, 77]
Infections (bacterial, viruses)	Image segmentation (watershed) algorithm	This technique segments WBCs in blood images, allowing for analysis of immune response to infections	[78, 79]
Autoimmune disorders	Ensemble methods (bagging, boosting)	Combining multiple models can improve the classification of WBCs involved in autoimmune responses, providing robust predictions	[80, 81]
Identifying autoimmune conditions	Neural networks	Deep neural networks help in detecting specific patterns in WBCs that indicate autoimmune conditions	[82]
Bone marrow disorders	Clustering algorithms (*K*-means, hierarchical)	Clustering techniques analyze the distribution of different WBC types in bone marrow samples, aiding in diagnosing disorders like myelodysplastic syndromes	[83]
HIV/AIDS	Classification trees	Trees classify immune cell types based on their counts, aiding in the management of HIV/AIDS and monitoring immune response	[84–86]
Hematological malignancies	CNNs for image analysis	CNNs are applied to classify and segment bone marrow cells, assisting in diagnosing hematological malignancies	[1, 70, 71, 87]
Predicting disease progression	Random forest	Useful in predicting disease progression based on the classification of WBC types in HIV-positive patients	[51, 68]
COVID-19 and related pneumonias	Deep learning (transfer learning)	Leveraging pre-trained networks to classify lung imaging data related to WBC response in COVID-19 pneumonia cases	[51, 67, 88]
Classifying WBCs related to COVID-19	Semi-supervised learning	Used in scenarios where labeled data is scarce, improving the classification of WBCs from unlabeled datasets related to COVID-19 studies	[48, 49, 51, 89–92]
Detect and count WBC	Computer vision (YOLOv5)	Utilized computer vision for detection and counting of WBC and achieved high level of accuracy and speed	[93]

The ML models developed by Gautam et al. [61] achieved high performance for blood cell classification. However, the results vary depending on the nature and diversity of the datasets used. Similarly, [9, 36, 54, 84, 94] showed that DL models can excel at tasks like blood cell classification and leukemia screening, surpassing traditional ML methods, while automatic classification of leukemic cells is effective with ML [91].

Another DS method used is KNN. Acharya and Kumar [95] effectively applied KNN in blood cancer detection, with solid results in terms of precision and recall, especially when combined with other methods like image processing (IP) [54]. Orth et al. [94] demonstrated that integrating with DL can improve WBC classification accuracy and speed [96], while Escobar et al. [93] employed YOLOv5 to achieve high accuracy and speed in WBC detection. Classification analysis is employed to categorize cell types based on images and classification of acid droplets from metastatic breast cancer patients. Other studies demonstrated that the algorithm enhances the classification of lymphocytes [97] and the detection of anemia and many other hematologic diseases [82]. Accurate detection of infection in blood streams and the detection of acute lymphoblastic leukemia or other types of blood cancer have been studied in the literature using AI models.

Clustering algorithms (e.g., *K*-means, hierarchical technique) are other functional DS methods utilized for WBC segmentation analysis, which are most effective in categorizing unknown groupings of comparable data into recognized groups [83, 92, 95]. Abnormal subgroups of blood cells with related clinical traits or medical conditions were segmented using AI and DS tools and techniques, such as ML and DL. Several studies focused on utilizing ML to predict or detect leukemia, and others addressed chronic granulomatous disease (CGD), a genetic disorder that affects the immune system, particularly the ability of white blood cells to kill bacteria and fungi [95, 98]. More use cases involving DS methods and WBC detection are available in Table 5.

CNNs are a type of deep learning model designed for image processing tasks. The typical architecture consists of extracting spatial features from WBC images using filters (kernels) and performing final classification based on extracted features. It is highly effective for feature extraction and thus can automatically learn morphological differences in WBCs. However, it requires large, labeled datasets and high computational power.

Random forest is an ensemble learning method that consists of multiple decision trees. The key components include an aggregation of bootstrapping called bagging that generates multiple decision trees trained on different subsets of the dataset. Each tree considers a random subset of features to reduce correlation. The final classification is based on the majority vote from all trees.

SVMs are supervised learning models that classify data by finding the optimal hyperplane that separates different classes. It transforms input features into a higher-dimensional space where they are more separable and optimizes the margin between different WBC classes. Unlike CNNs, SVMs require fewer samples for effective training and work best with handcrafted features thus is often combined with feature extraction methods like principal components analysis (PCA).

A recent study by Novia et al. (2023) [99] compared handcrafted feature-based methods with deep learning approaches such as DenseNet-169 for extracting color and shape features from segmented nuclei and cytoplasm white blood cell classification in porcine samples. Their findings showed that while handcrafted features achieved a competitive 91% accuracy with a non-linear SVM, the fine-tuned DenseNet-169 model achieved a slightly higher accuracy of 93%, demonstrating the overall superiority of deep neural networks in WBC classification tasks.

Over the years, AI technology and DS methods contribute significantly to the advancement of WBC detection research through auto mated image analysis, where machine learning algorithms and other analytic techniques are employed to efficiently analyze microscopic images of blood cells. The identification and classification of various types of blood cells are automated, helping to speed up diagnostics processes and allowing hematologists to focus on interpreting results. As such, diagnosis and classification of diseases related to WBCs by analyzing patient data patterns to identify specific abnormalities contribute to ensuring accurate and timely performance.

### Effectiveness of AI and DS Methods for WBC Detection, Segmentation, and Classification

This section evaluates the effectiveness of AI and DS methods for detecting, classifying, and segmenting WBC based on five criteria: accuracy, speed, robustness, interpretability, and clinical relevance. The results help to address the fourth research objective (RO4).

#### Accuracy

Many DS methods, such as DL techniques, CNNs, and transfer learning models (e.g., VGG16, ResNet), demonstrate high accuracy in WBCs’ classification tasks. Studies have reported accuracy rates often exceeding 90% in distinguishing between different types of WBCs [59, 67, 88].

Accuracy measures the overall correctness of a model, representing the proportion of correctly classified instances (both true positives and true negatives) out of the total samples. Many DS methods, such as DL techniques, CNNs, and transfer learning models (e.g., VGG16, ResNet), were reported to have high accuracy in WBCs’ classification tasks. Munshi and Navdeti [67] proposed a fully automated deep network classification system with an accuracy of 95%, which outperformed many performances in literature work. Using “ResNet50 CNN” architecture, Kutlu [1] obtained cell types of Lymphocyte determination with a 99.52% accuracy rate, monocyte with a 98.40% accuracy rate, basophil with a 98.48% accuracy rate, eosinophil with a 96.16% accuracy rate, and neutrophil with 95.04% accuracy rate. In a comprehensive review, Asghar et al. [100] highlighted papers with conventional ML models that have achieved up to 99% accuracy in WBC classification. However, the accuracy tends to decrease with smaller dataset sizes. Learning models, particularly those employing CNNs, have shown improved performance with larger datasets.

#### Robustness

DS methods are generally robust to variations in image quality, lighting conditions, and other factors that can affect WBCs’ imaging. Techniques like data augmentation help improve model generalization by training on diverse datasets. These DS methods can be fine-tuned to handle different datasets and enhance adaptability to various clinical settings and populations.

#### Interpretability

Generally, there is a challenge with DL models, such as the lack of explainability and interpretability. While DL models can achieve high performance, understanding the rationale behind decisions can be difficult, which is a concern in clinical applications where transparency is crucial. Emerging techniques such as the “gradient-weighted class activation mapping” (Grad-CAM) and “local interpretable model-agnostic” (LIME) are being developed to enhance interpretability by providing visual explanations of model decisions [80]. However, most studies often need to give a succinct interpretation of the models.

The Grad-CAM can be used to highlight regions of a blood smear image that are most important for classifying WBC types [80]. Since this technique generates heatmaps by computing the gradients of the output class concerning the final convolutional layers, it can highlight features that contribute most to the prediction. In hematology, Grad-CAM can visually show pathologists which parts of the cell were critical for the model’s decision. LIME operates by approximating the black-box DL model with an interpretable, local surrogate model that explains a single prediction. For WBC classification, LIME can perturb an image and observe how the changes affect the model’s prediction, helping identify which features, such as specific color patterns or shapes, are most influential for classification.

#### Clinical Relevance

As seen in most papers, DS methods have shown promise in augmenting the diagnostic capabilities of healthcare providers [59, 59, 72, 75]. By providing quantitative assessments of WBCs’ classifications, DS methods now support clinical decision-making and help monitor treatment responses.

#### Speed

Most of the papers evaluated in this review did not discuss the speed of processing the classification and segmentation of WBCs when applying DS methods. However, DS methods can facilitate real-time or near-real-time classification in general and of WBCs, which is crucial in clinical settings where timely diagnosis impacts patient outcomes. This aspect can benefit from further studies.

The application of AI and DS methods for WBCs’ detection shows excellent promise and effectiveness, especially in enhancing diagnostic accuracy and efficiency in clinical settings. However, challenges such as interpretability, generalizability, and the need for standardized protocols must be addressed to realize DS potential in healthcare fully. Continued research, validation, and collaboration between data scientists and medical professionals will be essential to optimize these systems for real-world applications.

#### Computational Efficiency

Despite the rapid advancement of AI models in clinical diagnostics, computational efficiency encompassing inference time, model size, and hardware resource demands remain underreported. Most studies emphasize diagnostic accuracy but provide limited information on inference speed or hardware requirements. High-latency or resource-heavy models may not be practical despite strong performance metrics in clinical settings where rapid decision-making is essential and computational resources are constrained. Therefore, future research should systematically report computational efficiency benchmarks, including inference times on standard clinical hardware, and explore model compression or lightweight architectures. Addressing these gaps ensures that AI models are effective, scalable, equitable, and ready for integration into diverse healthcare systems.

#### Critical Evaluation of AI and DS Methods for WBC Detection

A critical evaluation of AI and DS methods and techniques for WBC detection, segmentation, and classification further reveals the strengths, weaknesses, and limitations identified in the above section. Many studies utilizing the AI and DS methods and techniques reported high accuracy as the primary metric, which can be misleading, especially in imbalanced datasets where one class significantly outweighs the others. A model achieving 95% accuracy may still be ineffective if the disease prevalence is low (e.g., in leukemia detection, where the presence of abnormal cells might be < 5%). If a dataset has 95% normal WBCs and 5% leukemic WBCs, a model that always predicts “normal” will have 95% accuracy but 0% sensitivity, thus completely failing at detecting the disease.

##### Sensitivity (Recall or True Positive Rate)

Sensitivity measures the ability of a model to correctly identify positive cases, such as detecting abnormal WBCs. High sensitivity is crucial in clinical settings to ensure that diseased cells (e.g., leukemia cells) are not missing. Vaghashiya et al. [21] emphasizes the importance of sensitivity in early detection of hematological disorders. Rahman and Hasan [29] demonstrated a sensitivity of 96% in detecting acute lymphoblastic leukemia, highlighting the model’s effectiveness in minimizing missed diagnoses. Some studies focused only on sensitivity (recall) to highlight a model’s ability to detect diseased cells. High sensitivity without reporting specificity means the model may have a high false-positive rate, leading to unnecessary medical interventions. A leukemia classification model with 98% sensitivity but 60% specificity would correctly detect almost all true leukemia cases and falsely diagnose 40% of healthy patients.

##### Specificity (True Negative Rate)

Specificity measures the ability of a model to identify negative cases, minimizing false positives correctly. High specificity reduces the likelihood of healthy cells being incorrectly classified as diseased, which is critical to avoid unnecessary follow-up tests or treatments. In Suma (2022) [38], the specificity for normal cell classification reached 98%, indicating a low rate of false positives.

##### Precision (Positive Predictive Value—PPV)

Precision quantifies the accuracy of positive predictions, measuring the proportion of true positives among all positive predictions. High precision indicates a lower rate of false positives, which is crucial in minimizing alarm fatigue among clinicians. Kutlu (2020) [1] reported a precision of 97% for monocyte classification, ensuring that most identified monocytes were true positives, enhancing diagnostic reliability.

##### Precision-Recall (PR) Curve and F1-Score

The PR curve is particularly informative in cases of class imbalance, showing the trade-off between precision and recall across different thresholds. Area Under PR Curve (AUPRC) provides a single metric summarizing model performance. The *F*1-score balances precision and recall, which is especially valuable in medical applications where false positives and negatives have significant consequences. Hirani et al. (2024) reported an *F*1-score of 0.94 for neutrophil classification, indicating a balanced approach between precision and recall.

##### Receiver Operating Characteristic (ROC) Curve and AUC

Plots sensitivity vs. (1-specificity) across various threshold settings, providing insights into the trade-off between true positive and false positive rates. AUC values closer to 1 indicate superior model performance. An AUC > 0.90 is typically considered excellent in medical diagnostics. Capan et al. [11] showed an AUC of 0.92 for models predicting WBC abnormalities, suggesting reliable discrimination between normal and abnormal cells.

Incorporating a comprehensive set of metrics—accuracy, sensitivity, specificity, precision, PR curves, *F*1-score, and AUC-ROC—provides a more robust evaluation framework for AI models in WBC classification. Each metric offers unique insights into different aspects of model performance, particularly in assessing diagnostic reliability and clinical applicability. Future studies should prioritize these metrics to fully capture the strengths and limitations of AI and DS models in biomedical diagnostics, ensuring their safe and effective integration into clinical practice.

### Limitations of AI and DS Methods for WBC Detection, Segmentation, and Classification and Ethical Considerations

Despite the significant benefits of DS methods for WBC detection, segmentation, and classification, such as high accuracy, robustness, and clinical relevance, there are several limitations. Some key challenges include variability in image quality, dataset bias and limitations that hinder model generalizability, and ethical issues. These issues are limitations and challenges in this section, which further help to answer the fourth research objectives (RO4).

The quality variation in medical images stems from differences in microscope settings, staining methods, and imaging equipment, which can affect model accuracy and generalization [101]. The performance of DS models can be significantly affected by variability in image acquisition conditions. Differences in equipment, lighting, staining techniques, and microscope illumination can alter image contrast and affect model performance. Some studies utilize WBC image approximation. For example, Escobar et al. [93] utilized WBC sample images of pigs for the study, while the results become misleadingly generalizable, including in the human context.

Another critical challenge is dataset bias and limitations. These problems arise from various factors, including quality variation, imbalanced datasets, lack of diversity, and standardization. Imbalanced data sets are cases where certain classes (e.g., specific WBC types) are under or over-represented. Some WBC types (e.g., neutrophils) appear more frequently in datasets than others (e.g., basophils), leading to biased model predictions. Models trained on datasets from a specific population may not generalize well to other populations with different genetic or hematological characteristics. Differences in staining protocols, imaging equipment, and sample preparation techniques across laboratories can impact performance. The identified problems can lead to models that favor over or underrepresented classes, resulting in poor predictive performance for minority classes [102]. Further, the variations in staining intensity can lead to inconsistent cell morphology, making it harder for models to distinguish WBC subtypes. Differences in image resolution across datasets can impact image feature extraction, leading to misclassification. The presence of artifacts, blur, or occlusions in blood smear images can degrade classification accuracy [1, 7, 8, 103].

Most DS models are trained on specific datasets, and bias in these datasets can lead to reduced generalizability when applied to new patient populations or different laboratory settings. The lack of diversity in training datasets can lead to AI models that do not perform equally well across all patient populations, especially for underrepresented demographics (e.g., minority ethnic groups or rare conditions) [104, 105]. Also, most studies use proprietary, institution-specific, or private datasets, which limit comparability, reproducibility, and large-scale validation of DS methods. The lack of standardized datasets leads to inconsistent performance across studies.

Datasets may lack sufficient diversity in terms of race, age, gender, or disease variants, making AI systems not fully representative of the population. Factors such as the cost of the necessary technology, availability of skilled personnel, and infrastructure disparities, e.g., advanced AI systems might be more accessible in well-resourced urban centers, leaving rural or low-income areas at a disadvantage. These divide risks reinforcing the gap in healthcare outcomes between different socioeconomic groups. Some studies utilize WBC image approximation. For example, Escobar et al. [93] utilized WBC sample images of pigs for the study, while the results become misleadingly generalizable, including in the human context. Many models are trained on datasets that are not representative of a broader/global population, potentially leading to biased results. For instance, studies that rely on datasets from specific demographic groups, such as pigs (as seen in the work of Escobar et al. [93]), may yield results that do not generalize well to humans, let alone diverse human populations. Additionally, datasets may be skewed regarding race, age, or gender, leading to AI and DS models that perform poorly on underrepresented groups. This bias could perpetuate health disparities, particularly when diagnosis relies on AI-assisted tools.

The rapid deployment of AI in pathology requires robust ethical frameworks to guide its integration into clinical practice. Responsible AI deployment must prioritize transparency, accountability, and fairness. Addressing these biases must extend beyond the dataset to ensure that models are trained on diverse and representative samples while implementing mechanisms to audit AI decision-making processes and outcomes. Clinical practitioners must have the knowledge and tools to understand and explain AI-assisted diagnoses to patients, noting that AI can only complement and not replace human judgment. Furthermore, regulatory bodies must establish clear guidelines for developing, testing, and monitoring AI models to guarantee meeting high ethical standards before being deployed in healthcare settings. Medical images, particularly those used in WBC classification, contain sensitive patient information, and the use of these images raises significant concerns about data privacy. Medical data sharing, storage, and processing must adhere to strict regulatory standards such as the Health Insurance Portability and Accountability Act (HIPAA) in USA or the General Data Protection Regulation (GDPR) in Europe. However, despite these regulations, the large-scale aggregation of medical datasets, often including images and metadata, is susceptible to data breaches or potential misuse. For AI models to be deployed responsibly in clinical settings, systems must be designed with rigorous safeguards to protect patient privacy and ensure that data handling practices are transparent and secure.

## Conclusion and Recommendations for Future Research

This study conducted a literature analysis and evaluated AI and DS methods and novel algorithms employed for WBC classification and segmentation. These AI and DS methods include machine learning, big data analytics, deep learning, and novel/existing algorithms. Other techniques used are clinical decision support systems and visual analytics/visualization. These techniques effectively tackled the problems, improving performance, especially accuracy and efficiency. The reviewed literature focused mainly on detecting, identifying, classifying, and segmenting blood cells using WBCs for predicting and diagnosing leukemia. Other areas covered include hematological disorders like myelodysplastic syndromes and other blood-related cancers.

A major highlight in our evaluation involves uncovering the transformative impact of artificial intelligence in medical image analysis, while achieving high accuracy levels (most papers highlighted an accuracy score of more than 85%). However, ongoing challenges emphasize the importance of robust validation, addressing biases, and effectively integrating and adopting these technologies into real-world clinical practice. Continued research and collaboration between medical professionals and data scientists are pivotal for advancing these technologies and translating them into practical, impactful healthcare solutions.

While this study has demonstrated the effectiveness of AI and DS methods in WBC detection, segmentation, and classification, several areas remain unexplored. Future research could benefit from large language models to develop generative models for WBCs, focusing on enhancing model robustness, improving generalizability, and expanding diagnostic applications. Current models primarily rely on microscopic or blood smears. However, multimodal deep learning models, such as transformers and attention-based architectures, can be developed to process and interpret diverse WBC image information. WBC imaging conditions vary between different hospitals, laboratories, and devices. Generative models can help synthesize more realistic medical images, including blood smear images, to augment existing datasets and mitigate data scarcity. Generating diverse and high-quality samples can augment data availability, particularly for underrepresented classes or rare diseases, based on many past images observed. By expanding datasets, generative AI can improve the generalizability of WBC classification models, allowing them to perform better across diverse patient populations and varying image qualities.

Another recommendation for future research involves focusing on real-world applicability, developing training models using adaptation techniques to minimize performance drops across datasets from different sources. One of the major barriers to AI adoption in healthcare is the lack of interpretability. Most existing WBC datasets lack diversity in patient demographics, disease conditions, and imaging techniques. Integrating multiple data modalities, such as imaging data, clinical records, and lab results, can enrich the biological context of WBC classification. For example, combining blood smear images with patient demographics or clinical history could improve diagnostic accuracy and robustness, particularly in distinguishing between WBC types and identifying disease states that might not be evident from images alone. Further, genomic data integration with imaging could provide a deeper biological context for understanding WBC behavior and classification. By incorporating molecular profiles or genetic markers related to immune system function, future AI models could enhance their ability to distinguish between subtle differences in WBC morphology linked to specific genetic or genomic variations. These processes can significantly improve the model’s precision, especially in complex or rare cases where imaging alone is insufficient. Analyzing genomic data can help identify the genetic factors related to WBC functions, enhancing understanding of the basis of certain WBC disorders.

Finally, bridging the gap between AI research and clinical practice will require ongoing collaboration between data scientists, medical professionals, and regulatory bodies to ensure ethical, transparent, and effective AI deployment in hematology diagnostics. Such studies can also help in drug discovery for effective treatment through the discovery of new therapies for conditions like leukemia or autoimmune disorders affecting WBCs.

## Data Availability

The authors do not have permission to share the proprietary data used in this study.

## Abbreviations

AI: Artificial intelligence
ALL: Acute lymphoblastic leukemia
AML: Acute myeloid leukemia
CLL: Chronic lymphocytic leukemia
CNN: Convolutional neural networks
DL: Deep learning
DS: Data science
KNN: *K*-Nearest neighbors
RO: Research objective
SP: Scientific publications
SVM: Support vector machines
